# In vitro antioxidant and cholinesterase inhibitory activities of *Elatostema papillosum* leaves and correlation with their phytochemical profiles: a study relevant to the treatment of Alzheimer’s disease

**DOI:** 10.1186/s12906-018-2182-0

**Published:** 2018-04-05

**Authors:** A. S. M. Ali Reza, Mohammad Shahadat Hossain, Sharmin Akhter, Md. Rezanur Rahman, Mst. Samima Nasrin, Md. Josim Uddin, Golam Sadik, A. H. M. Khurshid Alam

**Affiliations:** 1 Department of Pharmacy, Faculty of Science and Engineering, International Islamic University Chittagong, Chittagong, 4318 Bangladesh; 2 Department of Applied Nutrition and Food Technology, Islamic University, Kushtia, 7003 Bangladesh; 3 Department of Pharmacy, University of Rajshahi, Rajshahi, 6205 Bangladesh

**Keywords:** Alzheimer’s disease, Cholinesterase inhibitors, *Elatostema papillosum*, Antioxidant activity

## Abstract

**Background:**

Alzheimer’s disease (AD), one of the major causes of dementia, is an overwhelming neurodegenerative disease that particularly affects the brain, leading to memory loss and impairment of language and judgment capacity. The aim of the present study was to investigate the antioxidant and anticholinesterase properties of the leaves of *Elatostema papillosum* (EPL) and correlate with their phytochemical profiles, which are relevant to the treatment of AD.

**Methods:**

The dried coarse powder of EPL was extracted with 80% methanol (EPL-M80) by cold extraction method. The resultant EPL-M80 was assessed for acetylcholinesterase (AChE) and butyrylcholinesterase (BChE) inhibitory activity by the Ellman method. The antioxidant activity was determined by DPPH (1, 1-diphenyl-2-picrylhydrazyl) and hydroxyl radical scavenging assays. Quantitative phytochemical (phenolic and flavonoid contents) analysis of endogenous substances in EPL-M80 was performed by standard spectrophotometric methods.

**Results:**

EPL-M80 significantly (*p* < 0.05) inhibited AChE and BChE activity with IC_50_ of 165.40 ± 4.01 and 213.81 ± 3.57 μg/mL, respectively in a dose-dependent manner. Additionally, EPL-M80 exhibited strong radical scavenging activity against DPPH (IC_50_ = 32.35 ± 0.68 μg/mL) and hydroxyl radical (IC_50_ = 19.67 ± 1.42 μg/mL) when compared to that of standards. EPL-M80 was found to be rich in phenolic (23.74 mg gallic acid equivalent/g of dry extract) and flavonoid (31.18 mg quercetin equivalent/g of dry extract) content. Furthermore, a positive correlation (*p* < 0.001) was observed between the total phenolics and antioxidant as well as the anticholinesterase potential.

**Conclusions:**

The marked inhibition of AChE and BChE, and potent antioxidant activity of the leaves of *Elatostema papillosum* highlight its potential to provide an effective treatment for AD.

## Background

Alzheimer’s disease (AD), one of the leading causes of dementia, is an overwhelming neurodegenerative disease that particularly affects brain function, resulting in memory loss and impairment of language and judgment capacity. A considerable proportion of the advanced age population suffer from AD and early-onset forms of the disease can affect the younger population too [1]. AD is characterised by the formation of senile plaques composed of amyloid beta protein (Aβ), the formation of neurofibrillary tangles (NFT) and substantial loss of both cholinergic and cortical neurons [2, 3]. It has been reported that loss of the neurotransmitter acetylcholine is responsible for the total dysfunction of cholinergic neurotransmission that is observed in AD and which accounts for cognitive deficits [4]. Acetylcholinesterase is the primary enzyme responsible for the breakdown of acetylcholine within synapses of the cerebral cortex. Consequently, acetylcholinesterase inhibitors can be employed for the treatment of AD [5, 6].

Reactive oxygen species (ROS) are generated within the body by multiple metabolic processes and through the effects of a range of external factors [7]. The aberrant accumulation of ROS has been found in several chronic diseases, including AD suggesting that ROS may contribute to the pathogenesis of these diseases by inducing oxidative stress [8, 9]. The antioxidative defense system functions to counteract the potentially damaging effects of oxidative stress by the production of antioxidants to eradicate excess oxidants [10, 11]. Previous studies have revealed that antioxidants have significant potential to reduce the symptoms and incidence of AD [12, 13]. However, the availablity of acetylcholinesterase inhibitors is limited to only a few members that are currently used only for symptomatic treatment and have several adverse effects [14–17]. Additionally, synthetic antioxidants have been utilized but have also exhibited adverse side effects that include liver damage and carcinogenesis [18]. Natural sources, especially plants, provide a diverse and largely untapped reservoir of substrates for drug discovery and offer great potential for the development of new cholinesterase inhibitors and antioxidants. Previous studies have already highlighted the potential of plants as vital sources for cholinesterase inhibitors and antioxidants [6, 19].

*Elatostema papillosum* Wedd. (*E. papillosum*) is a suberect herb (commonly known as Elya) that belongs to the Urticaceae family. Many Elatostema species are distributed in Africa, Asia, Australia and Oceania [20]. *E. papillosum* is found throughout China, Bhutan, India and Bangladesh. It was previously reported that crushed *E. papillosum* plants are used in traditional medicine for the treatment of hysteria and abdominal pain [21]. To the best of our knowledge, although there has been no reported pharmacological effect of *E. papillosum*; in our traditional medicine, the plant, has widely been used by local practitioner against various diseases, including hysteria [21], which is characterized by psychological disorder whose symptoms are closely associated with Alzheimer’s disease [22], encouraged us to carry out the pharmacological activity of this plant in this study. Therefore, the objective of this study was to assess the antioxidant and anticholinesterase activities of *E. papillosum* leaves (EPL), in order to determine its relevance for the treatment of AD.

## Methods

### Chemicals

Aluminium chloride, ammonium molybdate, ascorbic acid (AA), bicinchoninic acid, DPPH, Folin–Ciocalteu reagent, Tris-HCl and Triton X-100 were obtained from Sigma-Aldrich (Bangalore, India). Gallic acid (GA) was obtained from Wako Pure Chemical Company Ltd. (Osaka, Japan). 2-deoxy-D-ribose, thiobarbituric acid, (+)-catechin, 5,5-dithio-bis-(2-nitro) benzoic acid, acetylthiocholine iodide and donepezil were obtained from Sigma-Aldrich (Tokyo, Japan). Unless otherwise specified, all other chemicals were of analytical grade.

### Collection of plant

The EPL (leaves of *E. papillosum*) were collected from the Chittagong district, Bangladesh, and identified by Dr. Sheikh Bokhtear Uddin, taxonomist and professor, Department of Botany, University of Chittagong. A voucher specimen was submitted to the herbarium of the Department of Pharmacy, International Islamic University Chittagong, Chittagong, Bangladesh.

### Methanol extract

The EPL were dried at room temperature (RT) and crushed into fine powder to be used for extraction. Powdered leaves (500 g) were placed in a bottle and soaked in 80% methanol. The contents were sealed in the bottle for 7 days with occasional shaking and stirring. The whole mixture was filtered through cotton and Whatman No. 1 filter paper and the filtrate was concentrated with a rotary evaporator under reduced pressure at 50 °C to obtain 11.48 g of crude methanol extract of leaves of *E. papillosum* (EPL-M80).

### Experimental animals

Six to seven week-old Wistar albino rats weighing 150–200 g of both male and female were collected from International Center for Diarrheal Diseases Research, Bangladesh (ICDDRB) and housed in polypropylene cages under controlled conditions. The animals were exposed to alternative 12:12 h light and dark cycle at an ambient temperature of 26 ± 2 °C. Animals were allowed free access to drinking water and pellet diet, collected from ICDDRB, Dhaka. Rats were acclimatized for 7 days in the laboratory environment prior to the study.

### Ethics approval and consent to participate

The set of rules followed for rats experiment were approved by the Rajshahi University Animal Ethical committee (27/08/RUBCMB), according to governmental guidelines [23]. This research work was approved by Ethical Review Committee for rats of Research Cell of Rajshahi Medical College, Bangladesh (ref. RMC/ER/2010–2013/01).

### Euthanasia method

Compassion, professional ethics, and public sensitivity require that animals are euthanized humanely and appropriately under both planned and emergent situations. According to the 2013 AVMA Guidelines for the Euthanasia of Animals, intraperitoneal injection of ethanol is “acceptable with conditions” for use in rat [24]. Moreover, according to the method described by Allen-Worthington et al., 2015 [25], we applied 70% (*v*/v) ethanol in 0.9% sterile saline in the ventral chest region for getting deep anesthesia.

### Determination of total phenolic content

The total phenolic content was determined by the spectrophotometric method [26]. In brief, 1 mL of sample (1 mg/mL) was mixed with 1 mL of Folin-Ciocalteu’s phenol reagent. After 5 min of incubation at RT, 10 mL of a 7% Na_2_CO_3_ solution was added to the mixture followed by the addition of 13 mL of deionized distilled water and mixed thoroughly. The mixture was kept in the dark for 90 min at 23 °C, after which the absorbance was measured at 750 nm. The total phenolic content was determined from extrapolation of a calibration curve which was made by preparing gallic acid (GA) solution. The estimation of the phenolic compounds was carried out in triplicate. The total phenolic content was expressed as milligrams of gallic acid equivalents (GAE) per g of dried sample.

### Determination of total flavonoid content

The total flavonoid content was estimated by the aluminum chloride method described by Yang et al. [27]. The EPL-M80 (0.5 mL) was mixed with 2.5 mL of distilled water and 150 μL NaNO_2_ solution (5%). The contents were vortexed for 10 s and left at RT for 5 min. Then, 300 μL AlCl_3_ (10%), 1 mL NaOH (1 mM) and 550 μL of distilled water were added. The solution was mixed well and kept for 15 min at RT. The absorbance of each sample was measured at 510 nm. Catechin (CA) concentrations ranging from 31.25–500.00 μg/mL were prepared and the standard calibration curve was obtained. The total flavonoid content was calculated using standard CA calibration curve. The results were expressed as milligrams of catechin equivalents (CAE) per g of dry extract.

### Determination of DPPH radical scavenging activity

The free radical scavenging activity of the EPL-M80 was measured by in vitro DPPH assay according to the method described earlier [28, 29]. The stock solution was prepared by dissolving 24 mg DPPH with 100 mL methanol and stored at 20 °C until required. The working solution was obtained by diluting DPPH solution with methanol to attain an absorbance of about 0.98 ± 0.02 at 517 nm using the spectrophotometer. A 3 mL aliquot of this solution was mixed with 100 μL of the sample at various concentrations (10-100 μg/mL). The reaction mixture was shaken well and incubated in the dark for 15 min at RT. Then the absorbance was taken at 517 nm. The control was prepared as above without any sample. The scavenging activity was estimated based on the percentage of DPPH radical scavenged as the following equation:

$$ \%\mathrm{of}\ \mathrm{DPPH}\ \mathrm{radical}\ \mathrm{scavenging}\ \mathrm{activity}=\left[\left(\mathrm{control}\ \mathrm{absorbance}-\mathrm{sample}\ \mathrm{absorbance}\right)/\left(\mathrm{control}\ \mathrm{absorbance}\right)\right]\times 100 $$

### Determination of hydroxyl radical scavenging activity

Hydroxyl radical scavenging activity of the EPL-M80 was determined by the method described by Islam et al. [30]. The assay is based on the quantification of the degradation product of 2-deoxy-D-ribose by condensation with thiobarbituric acid (TBA). Hydroxyl radical was generated by the Fe^3+^ ascorbate-EDTA-H_2_O_2_ system (the Fenton reaction). In a final volume of 1 mL, the reaction mixture contained 2-deoxy-D-ribose (2.8 mM); KH_2_PO_4_-KOH buffer (20 mM, pH 7.4); FeCl_3_ (100 μM); EDTA (100 μM); H_2_O_2_ (1.0 mM); AA (100 μM); and various concentrations of the test sample or reference standard CA. After incubation for 1 h at 37 °C, 0.5 mL reaction mixture was added to 1 mL of 2.8% trichloroacetic acid, then 1 mL of 1% aqueous TBA was added, and the mixture was incubated at 90 °C for 15 min to develop the color. After it cooled, the mixture’s absorbance was measured at 532 nm against an appropriate blank solution. The percentage (%) of hydroxyl radical scavenging ability was calculated by using the following formula:

$$ \%\mathrm{of}\ \mathrm{hydroxyl}\ \mathrm{radical}\ \mathrm{scavenging}\ \mathrm{activity}=\left({\mathrm{A}}_{\mathrm{absorbance}\ \mathrm{of}\ \mathrm{control}}-{\mathrm{A}}_{\mathrm{absorbance}\ \mathrm{of}\ \mathrm{sample}}\right)/{\mathrm{A}}_{\mathrm{absorbance}\ \mathrm{of}\ \mathrm{control}}\Big)\times 100 $$

### Determination of cholinesterase inhibitory activity

The AChE inhibitory assay was performed according to the colorimetric method by Ellman et al. [31] with acetylthiocholine iodide as a substrate. For the enzyme source, rat brains were homogenized by a homogenizer with five volumes of ice-cold homogenization buffer (10 mM Tris-HCl (pH 7.2), which contained 1 M NaCl, 50 mM MgCl_2_ and 1% Triton X-100) and centrifuged at 10000 *g* for 30 min. The resulting supernatant was used as an enzyme source. All of the extraction steps were carried out at 4 °C. Protein concentration was determined by using a bicinchoninic acid kit (Sigma Co., St. Louis, MO, USA) with bovine serum albumin as a protein standard. The rates of hydrolysis by AChE were monitored spectrophotometrically. The EPL-M80 or standard (500 μL) was mixed with an enzyme solution (500 μL) and incubated at 37 °C for 15 min. Absorbance was taken at 405 nm immediately after adding Ellman’s reaction mixture (3.5-mL 0.5 mM acetylthiocholine, 1 mM 5, 5′-dithio-bis (2-nitro benzoic acid)) in a 50 mM sodium phosphate buffer (pH 8.0) to the above reaction mixture. Readings were repeated for 10 min at 2 min intervals to verify that the reaction occurred linearly. The blank reaction was measured by substituting saline for the enzyme. Donepezil was used as a positive control. The percentage inhibition of AChE activity was calculated using the following formula:

$$ \%\mathrm{of}\ \mathrm{inhibition}\ \mathrm{of}\ \mathrm{AChE}\ \mathrm{activity}=\left(\left({\mathrm{A}}_{\mathrm{absorbance}\ \mathrm{of}\ \mathrm{control}}-{\mathrm{A}}_{\mathrm{absorbance}\ \mathrm{of}\ \mathrm{sample}}\right)/{\mathrm{A}}_{\mathrm{absorbance}\ \mathrm{of}\ \mathrm{control}}\right)\times 100 $$

Assessment of BChE inhibition was performed as described above except that the enzyme solution was 50 μL and acetylthiocholine iodide was replaced by butyrylthiocholine iodide. Galantamine was used as positive control. The percentage inhibition of BChE activity was calculated using the same formula as mentioned above for AChE activity.

### Statistical analysis

The data were analyzed by one-way ANOVA followed by Dunnet’s test to estimate significant differences between the test and control groups with GraphPad Prism Data Editor for Windows, Version 6.0 (GraphPad Software Inc., San Diego, CA). Values were expressed as mean ± standard error of mean (± SEM). *p* < 0.05 and *p* < 0.01 were considered as statistically significant.

## Results

### In vitro cholinesterase enzyme activity

**Fig. 1:**
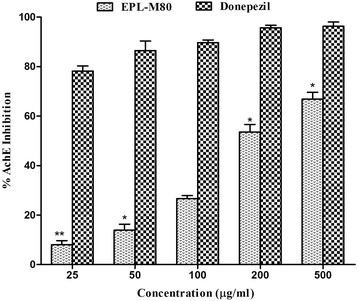
Percentage of inhibition of acetylcholinesterase activity at different concentration of EPL-M80 and the reference standard donepezil. Results are presented as mean ± SEM (*n* = 3). *p < 0.05 and **p < 0.01 denote significant difference compared to the control

**Fig. 2:**
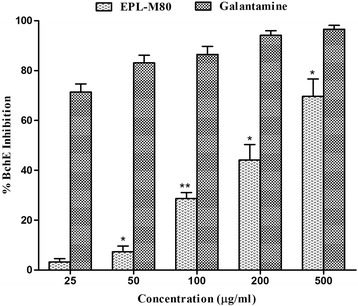
Percentage of inhibition of butyrylcholinesterase at different concentration of EPL-M80 and the reference standard galantamine. Results are presented as mean ± SEM (*n* = 3). *p < 0.05 and **p < 0.01 denote significant difference compared to the control

The critical role of cholinesterases in neural transmission makes them a key target of a large number of cholinesterase-inhibiting drugs relevant to the treatment of neurodegenerative disorders, including AD. To evaluate the potential of the EPL-M80 as an anti-AD drug, its AChE and BChE inhibitory activities were quantified. As shown in Figs. 1 and 2, the EPL-M80 showed significant (*p* < 0.05) AChE and BChE inhibitory effects when compared to the standards, and the cholinesterase inhibitory activity occurred in a dose-dependent manner. The AChE inhibitory activity of EPL-M80 was found to be 8.01 ± 1.68, 13.90 ± 2.43, 26.66 ± 1.21, 53.50 ± 3.09 and 66.82 ± 2.79 % at a concentration of 25, 50, 100, 200 and 500 μg/mL, respectively, with IC_50_ of 165.40 ± 4.01 μg/mL (Table 1). The BChE inhibitory activity of EPL-M80 was 3.24 ± 1.34, 7.31 ± 2.35, 28.67 ± 2.39, 44.14 ± 6.19, 69.70 ± 6.92 % at a concentration of 25, 50, 100, 200 and 500 μg/mL, respectively, with IC_50_ of 213.81 ± 3.57 μg/mL. In this study, donepezil and galantamine were used as reference standards. The IC_50_ of standard donepezil and galantamine against AChE and BChE were 17.01 ± 0.33 μg/ml and 19.64 ± 0.66 μg/mL, respectively (Table 1).

**Table 1:** IC_50_ values of EPL-M80 and standards in radicals scavenging and enzymes inhibitory activity assays

IC_50_s (μg/mL)				
Samples	DPPH radical scavenging	Hydroxyl radical scavenging	AChE	BChE
(+)-Catechin	2.32 ± 0.57^a^	8.08 ± 0.73	-	-
Donepezil	-	-	17.01 ± 0.33	-
Galantamine	-	-	-	19.64 ± 0.66
EPL-E80	32.35 ± 0.68	19.67 ± 1.42	165.40 ± 4.01	213.81 ± 3.57

^a^Each value is expressed as mean ± SEM (*n* = 3). EPL-M80 represents methanol extract of leaves of *E. papillosum*

### DPPH free radical scavenging activity

**Fig. 3:**
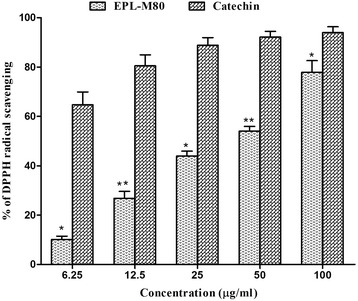
DPPH radical scavenging activity at different concentrations of EPL-M80 and the reference standard catechin. Results are presented as mean ± SEM (*n* = 3). *p < 0.05 and **p < 0.01 denotes significant difference compared to the control

The in vitro antioxidant activity of the EPL-M80 was measured in comparison to the standard antioxidant, CA. However, the percentage of DPPH radical scavenging activity occurred in a dose-dependent manner. The EPL-M80 displayed higher scavenging activity (77.84 ± 4.75%) with IC_50_ of 32.35 ± 0.68 μg/mL than the standard CA (93.98 ± 2.37%) with IC_50_ of 2.32 ± 0.57 μg/mL at the concentration of 100 μg/mL (Fig. 3 and Table 1), suggesting that EPL-M80 has the capacity to reduce OS caused by free radicals.

### Hydroxyl radical scavenging activity

**Fig. 4:**
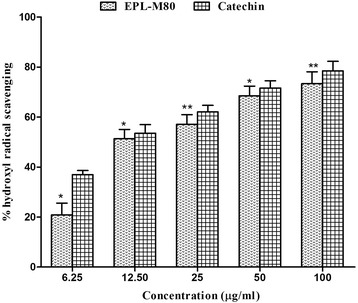
Hydroxyl radical scavenging activity at different concentrations of EPL-M80 and the reference standard catechin. Results are presented as mean ± SEM (*n* = 3). *p < 0.05 and **p < 0.01 denotes significant difference compared to the control

The hydroxyl radical is the major reactive oxygen species responsible for lipid oxidation and potentially severe biological damage. This assay shows how EPL-M80 and the standard CA inhibit hydroxyl radical-mediated deoxyribose degradation generated in a Fe^3+^ − ascorbate - EDTA - H_2_O_2_ system (the Fenton reaction). The hydroxyl radical scavenging capacity of the EPL-M80 and the standard CA were 73.31 ± 4.17% with IC_50_ of 19.67 ± 1.42 μg/mL) and 78.47 ± 3.81% with IC_50_ of 8.08 ± 0.73 μg/mL), respectively (Table 1). The results show EPL-M80 possesses similar capacity of hydroxyl radical scavenging to the standard CA (Fig. 4), suggesting EPL-M80 could provide a major source of antioxidant. Moreover, the ability of EPL-M80 to quench hydroxyl radicals might be of direct relevance to the prevention of lipid peroxidation.

### Total phenolic content

The total phenolic content of EPL-M80 was ascertained by the method of Folin ciocalteu and calculated as gallic acid equivalent (GAE). The phenolic content of the EPL-M80 was 23.74 mg GAE /g of dry extract (Table 2).

**Table 2:** Polyphenol contents of EPL-M80

Polyphenols	EPL-M80
Phenolics^a^	23.74 ± 0.04^1^
Flavonoids ^b^	31.18 ± 0.01

NB: ^1^Each value is the average of three analyses ± standard deviation. a and b expressed in terms of GAE and CAE, respectively (mg of GA and CA /g of dry extract, respectively). EPL-M80 represents methanol extract of leaves of *E. papillosum*

### Total flavonoid content

The aluminum chloride colorimetric method was adopted for the assessment of total flavonoid content. The content of total flavonoids was expressed as catechin equivalent (CAE). The flavonoid content of the EPL-M80 was 31.18 mg CAE/ g of dry extract (Table 2).

### Correlation and regression of phenolic contents with antioxidant and anticholinesterase potential

Table 3 shows the correlation and regression (*p* < 0.001) of phenolic contents with respect of antioxidant and anticholinesterase potential. A significant correlation (*p* < 0.001) was observed for EPL-M80.

**Table 3:** Correlation coefficients between the total phenolic contents and antioxidant as well as cholinesterase inhibitory activity of EPL-M80

	Total Phenolic Contents (Correlation R^2^)			
Assays	DPPH	HRSA	AChEI	BChEI
EPL-M80	^1^0.998^**^	0.969^**^	0.978^**^	0.855^*^

^1^Each value is represented as mean ± SD (*n* = 3). * indicates significance at ^*^P < 0.05, ^**^P < 0.01. DPPH = 2,2-diphenyl-1-picrylhydrazyl, HRSA = hydroxyl radical scavenging assay, AChEI = Acetylcholinesterase inhibition and BChEI = Butyrylcholinesterase inhibition. EPL-M80 represents methanol extract of leaves of *E. papillosum*

## Discussion

Alzheimer’s disease (AD) is a chronic neurodegenerative disorder characterized by the progressive impairment of memory, cognition and behavior that usually exhibits a slow onset before worsening over time and ultimately leading to death. The causes of AD are poorly understood [32], although several etiological factors, such as genetic abnormalities [33], history of head injuries, environmental factors, general lifestyles [34], depression or hypertension [32], deposition of extracellular *β*-amyloid protein (A*β*) and microtubule associated tau protein [33] in the brain, and cholinergic dysfunction have all been implicated in AD. At present, there are no drugs available that are capable of curing Alzheimer’s disease or any of the other common types of dementia, but two conceptual approaches for the treatment of AD have been developed. The first approach consists of treatment to avert the onset of the disease by sequestering the primary progenitors in order to prevent the development of AD. The second approach involves symptomatic treatment of the tertiary cognitive symptoms of the disease to protect against further cognitive decline [35]. However, the implementation of appropriate treatment strategies depends on the severity of the disease and the specificity of each individual case. Currently, only three cholinesterase inhibitors such as donepezil, galantamine, rivasigmine and a patial NMDA receptor antagonist, memantine, are the Food and Drug Administration (FDA) approved drugs to treat AD. The purpose of cholinesterase inhibitors is to inhibit the degradation of acetylcholine within synapses, resulting in increased amount of acetylcholine. Memantine, which is a partial NMDA receptor antagonist, protects neurons from glutamate-induced excitatory damages [1]. These strategies represent the only current treatments available for AD. The cholinesterase inhibitors employed in current treatments are limited and they show a wide variety of toxic effect including insomnia, anorexia, diarrhea, fatigue, nausea, gastrointestinal disorders and cardiovascular disorders [1, 2]. Therefore, researchers have focused their attention towards the discovery of new drugs from natural sources such as plants, which have enormous potential for the treatment of AD. In this study, we have discovered the capacity of EPL-M80 to significantly inhibit AChE and BChE activities in a dose-dependent manner (Figs. 1 and 2). The inhibition of cholinesterases was found to be better compared to other medicinal plants including *Andrographis paniculata*, *Nelumbo nucifera*, *Myrstica fragrans*, and *Aegle marmelos* [2, 7] suggesting EPL-M80 extract as an effective cholinesterase inhibitor and can be used in the treatment of AD [5].

Accumulating evidence suggests that brain tissues in AD patients are exposed to OS during the development of the disease. The biological damage caused by OS includes protein oxidation, lipid oxidation, DNA oxidation and glycoxidation [36] which are closely associated with the development of AD, cancer, diabetes etc. [37]. OS is generally characterized by an imbalanced production of ROS and reactive nitrogen species (RNS). OS is initiated by free radicals such as the superoxide anion radical (O_2_^*•−*^), hydrogen peroxide (H_2_O_2_), the hydroxyl radical (*•*OH), singlet oxygen (^1^O_2_), alkoxyl radicals (RO*•*) and peroxyl radicals (ROO*•*), which have a tendency to become stable through electron pairing with biological macromolecules like proteins, lipids and DNA in healthy human cells. This tendency has led to the assertion that OS contribute to the pathogenesis of numerous human degenerative diseases [38, 39]. Antioxidative defense systems act to remove ROS and prevent cellular damage by quenching free radicals, thereby protecting against diseases such as AD [40]. Such defense systems play a vital role in protecting living organisms from the damaging effects of free radical attacks, however, extensive biological damage can occur when the rate of free radical generation exceeds the capacity of this defensive system, leading to elevated ROS levels [41]. Elevated levels of ROS contribute to the pathogenesis observed during the course of AD. Therefore, we quantified the scavenging potential of EPL-M80 using the DPPH bleaching assay for antioxidant activity [42]. In this assay, the extent of color change is proportional to the potential and concentration of antioxidant activity, conferred by the hydrogen donating ability [43]. In our study, EPL-M80 showed high scavenging percentage of DPPH, reflecting its potent antioxidant activity (Fig. 3). The hydroxyl radical is an extremely damaging ROS formed by successive monovalent reduction of dioxygen (O_2_), capable of initiating lipid peroxidation which results in severe cell damage in vivo [44]. The short-lived hydroxyl radical is particularly damaging to the polyunsaturated fatty acid of cell membrane phospholipids with harmful effect to the cell [45]. Hydroxyl radicals are generated in the biological system by the Fenton reaction with subsequent degradation of deoxyribose to TBARS which generates a pink chromogen on heating at low pH with TBA [46]. In our investigation, EPL-M80 showed promising hydroxyl radical scavenging activity and was capable of protecting deoxyribose in a dose-dependent manner. There is evidence that the hydroxyl radical activity of the extract is directly proportional to its antioxidant activity [47] (Fig. 4). In biological systems, aberrant production of free radicals lead to extensive tissue and bio-molecules damage, which in turn cause a multitude of degenerative diseases [8, 48]. Phenolic and flavonoid compounds represent classes of antioxidant capable of acting as free radical scavenger [49, 50] to prevent cellular damage. In our study, we detected a cumulative increase of phenolic and flavonoid contents with increasing concentration of EPL-M80, suggesting that EPL-M80 is capable of reducing the risk of various degenerative diseases including AD, by eliciting antioxidative activities to prevent OS damage (Table 2). Previous studies also revealed that flavonoids act as free radical scavengers of many oxidizing species [51]. Total phenolic contents of EPL-M80 showed significant and strong positive correlation (*p* < 0.001) with antioxidants (DPPH ^*·*^ and ^*·*^ OH) and anticholinesterase (AChE and BChE) potential (Table 3). These results suggest a potential role of the polyphenolic constituents of EPL-M80 in free radical neutralization and inhibition of ChE activity. Further studies are warranted to isolate and characterize the active polyphenol compound that may be used as a candidate drug in AD.

## Conclusion

To the best of our knowledge, this is the first report describing the cholinergic inhibitory and antioxidant activities of *Elatostema papillosum*. However, further testing in an animal model of Alzheimer’s disease is needed to clarify the in vivo effectiveness of this plant.
